# Assessment of the gastrointestinal microbiota using 16S ribosomal RNA gene amplicon sequencing in ruminant nutrition

**DOI:** 10.5713/ab.22.0382

**Published:** 2023-01-24

**Authors:** Minseok Kim

**Affiliations:** 1 Division of Animal Science, Chonnam National University, Gwangju 61186, Korea

**Keywords:** Amplicon Sequencing, Gastrointestinal Tract, Microbiota, 16S rRNA Gene, Ruminant Nutrition

## Abstract

The gastrointestinal (GI) tract of ruminants contains diverse microbes that ferment various feeds ingested by animals to produce various fermentation products, such as volatile fatty acids. Fermentation products can affect animal performance, health, and well-being. Within the GI microbes, the ruminal microbes are highly diverse, greatly contribute to fermentation, and are the most important in ruminant nutrition. Although traditional cultivation methods provided knowledge of the metabolism of GI microbes, most of the GI microbes could not be cultured on standard culture media. By contrast, amplicon sequencing of 16S rRNA genes can be used to detect unculturable microbes. Using this approach, ruminant nutritionists and microbiologists have conducted a plethora of nutritional studies, many including dietary interventions, to improve fermentation efficiency and nutrient utilization, which has greatly expanded knowledge of the GI microbiota. This review addresses the GI content sampling method, 16S rRNA gene amplicon sequencing, and bioinformatics analysis and then discusses recent studies on the various factors, such as diet, breed, gender, animal performance, and heat stress, that influence the GI microbiota and thereby ruminant nutrition.

## INTRODUCTION

The gastrointestinal (GI) microbes play an important role in the digestion, performance, and health of ruminants. Within the GI microbes, the ruminal microbes are the most important in ruminant nutrition because they can digest and convert various feeds containing cellulose, hemicellulose, starch, protein, and lipid to volatile fatty acids (VFAs) and microbial proteins [1,2]. The small intestine plays an important role in post-ruminal digestion, and the microbial biomass and diversity in the small intestine are low due to the short transit time [3]. There are only a few studies on the microbiota in the small intestine of ruminants [4], but the results have shown that the microbiota composition in the small intestine differs from that in the rumen and large intestine [5]. Like the ruminal microbes, the microbes in the large intestine can also ferment nutrients escaping the small intestine and produce VFAs, major end-products important for maintaining gut health. Many ruminant nutritionists and microbiologists have focused their research efforts on identifying strategies for maintaining optimal GI fermentation and improving nutrient utilization efficiency [1,6].

Initially, GI microbes, particularly ruminal microbes, were assessed using culture-dependent methods, which contributed to understanding the functions and metabolisms of GI microbes. However, it became evident that only a small portion of the GI microbes could be isolated using this approach on the standard culture media used in the laboratory [2]. By contrast, because the 16S rRNA gene can serve as a phylogenetic marker to analyze microbial taxa, 16S rRNA gene sequencing can be used to identify unculturable GI microbes in ruminants [6]. As traditional methods, clone library construction and denaturing gradient gel electrophoresis (DGGE) analysis of 16S rRNA gene amplicons, followed by Sanger sequencing, can reveal the composition of the GI microbiota [2,7]. However, because of the low depth of microbial diversity analyzed by these methods, minor taxa cannot be detected using these two methods. Since amplicon sequencing of 16S rRNA genes was developed, many nutritional studies have assessed the microbial diversity of the GI microbiota in great depth [6].

The objective of this review is to discuss the association between the GI microbiota composition and various factors, such as diet, breed, gender, feed efficiency, marbling score, and heat stress, using 16S rRNA gene amplicon sequencing to improve the understanding of ruminant nutrition. In addition, some research challenges for nutrition studies of ruminants are discussed.

## 16S rRNA GENE AMPLICON SEQUENCING

**Figure 1:**
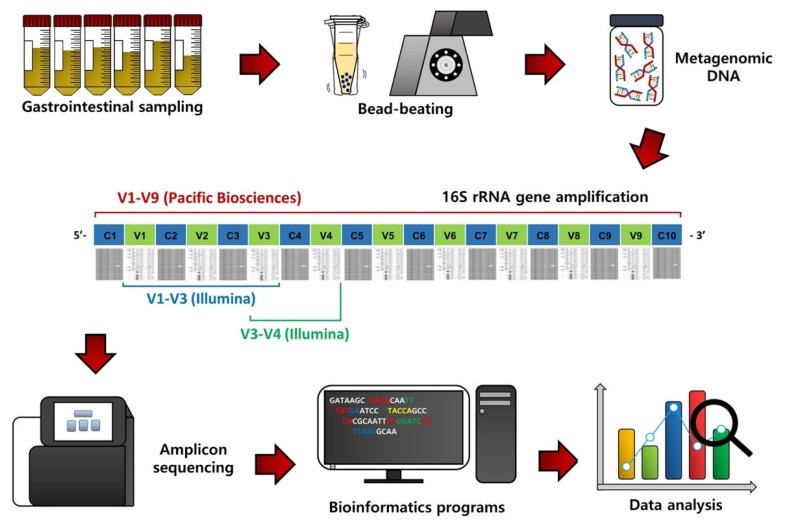
Flowchart outlining 16S rRNA gene amplicon sequencing, bioinformatic procedures, and data analysis of gastrointestinal content samples of ruminants.

To assess the GI microbiota in ruminants, the first procedure is GI content sampling and metagenomic DNA extraction (Figure 1). Contents of the rumen can be collected via a ruminal cannula or the stomach tube method [8,9], while fecal samples can be collected by rectal grab using a clean glove [10]. Contents of other segments of the GI tract, such as the duodenum, jejunum, ileum, cecum, and colon, can be collected from animals after sacrifice [5]. The bacterial metagenomic DNA can be extracted from the collected samples, usually using the bead-beating method, which can improve DNA yield [11].

From the extracted metagenomic DNA, 16S rRNA gene amplicons can be obtained using universal primers and subsequentially sequenced using a next-generation sequencing system (Figure 1) [6]. Initially, the 454 Genome Sequencer FLX system (Roche, Branford, CT, USA) was used for 16S rRNA gene amplicon sequencing, but this sequencer is no longer used due to low sequence reads and the high cost. Instead, most microbiota studies use the Illumina MiSeq/HiSeq sequencing platform (San Diego, CA, USA) because of high sequence reads and the low cost compared to the 454 Genome Sequencer FLX system. The Pacific Biosciences sequencing system (Menlo Park, CA, USA) is also used to sequence nearly full-length 16S rRNA gene amplicons because it can provide more accurate phylogenetic resolution, but the cost is still high.

Bioinformatics and data analyses are conducted on the resulting sequence data to assess the GI microbiota in ruminants (Figure 1). The QIIME software package is one of the most popular bioinformatics programs for sequence processing, such as sequence assembling, demultiplexing, denoising with quality filtering, and chimeric sequence detection [12]. Operational taxonomic units (OTUs) or amplicon sequencing variants can be classified based on the pre-trained reference databases, such as Greengenes and Silva, using the naïve Bayesian taxonomic classifier [13]. Alpha diversity, such as species richness, evenness, phylogenetic diversity, Shannon’s index, and Simpson’s index, can be used to evaluate microbiota diversity, while beta diversity based on principal coordinates analysis can be assessed to compare microbiota dissimilarities among treatment groups. In addition, from the 16S rRNA gene sequence data, functional features can be predicted using the Phylogenetic Investigation of Communities by Reconstruction of Unobserved States (PICRUSt) method [14].

## ASSESSMENT OF RUMINAL MICROBIOTA

Ruminal microbiota digest and ferment various feeds that are subsequently utilized by the host and thus have a crucial role in ruminant nutrition. Many studies have used traditional 16S rRNA gene-based methods, such as clone library construction [15], quantitative real-time PCR [16], and phylogenetic microarray [6], to evaluate various factors, such as diet, breed, gender, age, and geographic region [2], affecting the ruminal microbiota composition of various ruminant breeds. However, these traditional methods with low sequence reads cannot detect minor ruminal microbiota because of the low depth of percentage coverage of the microbial diversity. Since 16S rRNA gene amplicon sequencing producing high sequence reads was first used for microbiota studies, various factors affecting the ruminal microbiota composition have been assessed at an improved resolution of the microbial diversity in many nutritional studies.

### Comparison of rumen content sampling techniques

In ruminant nutrition, collecting samples of the rumen contents from ruminants allows an analysis of the rumen microbiota, digestibility, and fermentation parameters. For rumen content sampling, rumen cannulation has been used as the standard method in many studies [17]. However, its disadvantage is the need for a surgical procedure for cannulation, and the number of rumen-cannulated ruminants that can be used in an experiment is limited. As an alternative method, the stomach tube method is attractive because it enables multiple collections of the rumen contents from many ruminants. Therefore, the stomach tube method is advantageous for increasing the statistical power of the analysis [9].

Although previous studies used the traditional DGGE method to compare the ruminal microbiota between the cannulation and stomach tube method, the coverage depth of the rumen microbial diversity analyzed in these studies was limited [8,18,19]. Next-generation sequencing of 16S rRNA gene amplicons was used to assess the feasibility of the stomach tube method as an alternative to standard cannulation procedures. The cannulation and stomach tube methods gave similar results for the ruminal microbiota composition of Holstein and Jersey cattle [20]. Our recent study also indicated that the ruminal microbiota collected from Korean native Hanwoo cattle was not affected by the two different sampling methods [9]. In this context, 16S rRNA gene amplicon sequencing of the ruminal microbiota helped identify whether the stomach tube was a feasible alternative to the standard cannulation method in the field of ruminant nutrition.

### Diet and ruminal microbiota

Traditional 16S rRNA gene-based techniques, such as clone library construction and DGGE, have been used to assess how the rumen microbiota composition is affected by dietary changes in ruminants (e.g., [15]). However, these traditional methods detected only dominant microbes and represented only a small portion of the ruminal microbial communities [2]. Therefore, results produced by traditional methods may be biased.

High-resolution characterization of the microbial diversity using 16S rRNA gene amplicon analysis has been conducted to explore the effects of dietary changes on the composition of the ruminal microbiota. Some *in vitro* rumen fermentation studies evaluated the effects of different levels of additives on the ruminal microbiota composition (e.g., [21,22]) (Table 1). For example, Petri et al [21] evaluated the effects of different combinations of alkaloids, minerals, vitamins, prebiotics, and tannins on rumen microbiota, and these combinations increased the phylum *Bacteroidetes* and decreased the genus *Pyramidobacter*. Zhang et al [22] showed that a diet supplemented with grape seed procyanidin increased the abundance of *Methanomassiliicoccus* and decreased the abundance of *Methanobrevibacter* in the rumen. Other *in vitro* studies assessed the effects of different levels of feed ingredients on the ruminal microbiota composition (e.g., [23]). For example, when analyzing the effects of different levels of bakery by-products as a feed ingredient on rumen microbiota, Humer et al [23] found that the inclusion of bakery by-products increased the genera *Prevotella*, *Roseburia*, and *Megasphaera* (Table 1). Cui et al [24] assessed whether the ruminal microbiota composition was affected by different levels of energy and protein in the diet with the same ingredients (Table 1). In this study, the family *Prevotellaceae* and the genus *Butyrivibrio* were increased by the low energy level diet. In this context, 16S rRNA gene amplicon sequencing will remain a useful tool in future nutritional studies for assessing the effect of dietary interventions on the ruminal microbiota.

### Breed and ruminal microbiota

Host breed is another factor affecting the ruminal microbiota composition of ruminants. Recent studies showed that the ruminal microbiota composition analyzed by 16S rRNA gene amplicon sequencing was different between Holstein and Jersey cattle breeds fed the same diet [17,20] and was associated with different methane emissions from the rumen [17]. Differences have also been observed in the ruminal microbiota composition among Angus, Charolais, and Kinsella composite hybrid breeds (Table 1) [25]. Similarly, our recent study demonstrated that the overall microbiota composition of the rumen differed between Korean native brown Hanwoo and Jeju black cattle fed the same diet at the same farm [26]. Particularly, cellulolytic *Ruminococcus* was greater in brown Hanwoo cattle than in Jeju black cattle (Table 1). Host genetics can affect heritable rumen microbial features, and different breeds may shape distinct ruminal microbiota due to genetic influence [27]. Therefore, selective breeding may be one strategy to manipulate the ruminal microbiota composition [25].

### Gender and ruminal microbiota

A recent study of the ruminal microbiota composition in 709 beef cattle identified gender as one of the factors affecting the ruminal microbiota composition (Table 1) [27]. Another study showed that the ruminal microbiota composition differed between different genders of Tibetan goats (Table 1) [28]. Gut microbiota in humans was changed after male castration [29], indicating that different sex hormones could lead to microbial differences between genders [30]. In addition, body mass index (BMI) has been reported to be associated with the gut microbiota in humans [30]. The BMI was significantly associated with the gut microbiota in females, whereas it was not associated with the gut microbiota in males [31]. As shown in humans, BMI may differentially influence the ruminal microbiota between different genders.

### Feed efficiency and ruminal microbiota

Feed accounts for the largest portion of total cost in the beef industry, so improving feed efficiency is important to increase profitability in animal production. Although some studies evaluated the association between feed efficiency and the ruminal microbiota using the traditional DGGE method [32,33], the use of 16S rRNA gene amplicon sequencing has improved the depth of percentage coverage of microbial diversity for assessing and comparing the ruminal microbiota between high- and low-feed efficiency groups in beef cattle (Table 1) [34]. The results showed that the phylum *Firmicutes* and the families *Lachnospiraceae* and *Veillonellaceae* were more abundant in the high-efficiency group than in the low-efficiency group. At the genus level, *Acidaminococcus* was more abundant in the high-efficiency group than in the low-efficiency group, whereas *Anaerovibrio* was more abundant in the low-efficiency group than in the high-efficiency group. In addition, some OTUs assigned to the genus *Prevotella* were more abundant in the high-efficiency group than in the low-efficiency group. Since this study, many studies on the association between feed efficiency and the ruminal microbiota have been conducted in cattle [25,27,35] and sheep [36,37]. Li et al [25] indicated that the phylum *Firmicutes* was more abundant in the high-efficiency group than in the low-efficiency group, while the genera *Succiniclasticum*, *Moryella*, and *Blautia* were more abundant in the high-efficiency group than in the low-efficiency group in Charolais cattle (Table 1). Conversely, in Kinsella composite hybrid cattle, the genera *Butyrivibrio* and *Desulfovibrio* were more abundant in the high-efficiency group than in the low-efficiency group, whereas *Shuttleworthia*, *Desulfobulbus*, and *Mitsuokella* were more abundant in the low-efficiency group than in the high-efficiency group. Li et al [27] showed that the ratio of *Firmicutes* to *Bacteroidetes* was positively correlated with feed efficiency in beef cattle (Table 1). Paz et al [35] reported that OTUs assigned to the families *Prevotellaceae*, *Spirochaetaceae*, *Paraprevotellaceae*, *Veillonellaceae*, and *Lachnospiraceae* were more abundant in the high-efficiency groups than in the low-efficiency group, while *Prevotellaceae* OTUs were more or less abundant in the high-efficiency group than in the low-efficiency group in steers (Table 1). In heifers, one OTU assigned to the family *Victivallaceae* was more abundant in the high-efficiency group than in the low-efficiency group, whereas OTUs assigned to the families *Prevotellaceae* and *Fibrobacteraceae* were less abundant in the high-efficiency group than in the low-efficiency group. Our recent study assessed the association between feed efficiency and the ruminal microbiota in Hanwoo cattle [38]. Taxa abundant in high-efficiency ruminants may serve as potential biomarkers of high-feed efficiency and provide strategies to improve feed efficiency through the manipulation of the ruminal microbiota.

### Marbling score and ruminal microbiota

In the beef industry, increasing the marbling content is important to increase economic benefits. Our recent study assessed the association between the marbling score and the ruminal microbiota in Hanwoo cattle with a genetically high-marbling content [39]. In this study, Hanwoo steers belonging to either a high-marbling score group or a low-marbling score group were selected for comparison. Results showed that the overall ruminal microbiota composition differed between these two extreme groups, and the lipid metabolism pathways were enriched in the high-marbling score group in functional prediction. Taxa identified in high-marbled cattle (Table 1) may be targeted to increase marbling content through the manipulation of the ruminal microbiota.

### Heat stress and ruminal microbiota

16S rRNA gene amplicon sequencing has provided valuable insight into the impact of heat stress on the ruminal microbiota composition of cattle. Correia Sales et al [40] indicated differences in the ruminal microbiota composition between thermoneutral (24°C) and heat-stressed groups (34°C) of Nellore cattle; in particular, heat stress decreased the relative abundance of fibrolytic bacteria. In another study, lactate-producing bacteria decreased, and acetate-producing bacteria increased in Holstein dairy cattle exposed to heat stress (34°C) compared to the thermoneutral (24°C) group [41]. Our recent study assessed the ruminal microbiota composition in Hanwoo cattle exposed to acute heat stress in climate-controlled chambers [42]. The results showed that after the environmental temperature of 15°C was raised to 35°C at 60% humidity, the fibrolytic bacteria decreased, whereas the lactate-producing bacteria increased (Table 1). In cattle exposed to heat stress, increased lactate production reduces the ruminal pH and, subsequently, the abundance of fibrolytic bacteria, which are sensitive to low pH [43]. These 16S rRNA gene amplicon analyses may contribute to developing new feeding strategies to improve the adaptability of ruminants and maintain a normal ruminal microbiota composition under heat stress [44].

## ASSESSMENT OF INTESTINAL MICROBIOTA

While most of the microbiota studies published to date have focused on the rumen or feces of ruminants, some studies have assessed the microbiota in the small and large intestine of ruminants. Myer et al [45] showed that the jejunal microbiota composition was associated with feed efficiency in beef cattle; specifically, OTUs assigned to *Butyrivibrio* were more abundant in the efficient group than in the inefficient group (Table 1). Liu et al [4] collected samples of the duodenal, jejunal, and ileal contents to evaluate the association between feed efficiency and the small intestine microbiota in Angus cattle (Table 1). In this study, the families *Lachnospiraceae*, *Ruminococcaceae*, and *Christensenellaceae* were more abundant in the efficient group than in the inefficient group in the duodenum [4]. The family *Lachnospiraceae* was more abundant in the efficient group than in the inefficient group in the jejunum, while the families *Ruminococcaceae* and *Christensenellaceae* were more abundant in the efficient group than in the inefficient group in the ileum [4]. Other researchers investigated the association between the microbiota of the large intestine (e.g., cecum and colon) with feed efficiency in cattle [46,47]. Freetly et al [5] assessed the ruminal, duodenal, jejunal, ileal, cecal, and colonic microbiota to investigate their associations with animal performance in beef cattle (Table 1). In the duodenum, OTUs assigned to *Lachnospiraceae*, *Ureibacillus*, *Bacillus*, and *Prevotella* were more abundant in the inefficient group than in the efficient group, while the reverse held true for one OTU assigned to *Lachnospiraceae* [5]. In the jejunum, OTUs assigned to *Butyrivibrio*, *Dialister*, *Desulfovibrio*, *Agrobacterium*, and *Ochrobactrum* were more abundant in the efficient group than in the inefficient group, whereas OTUs assigned to *Mogibacterium*, *Shuttleworthia*, *Lactobacillus*, *Corynebacterium*, and *Atopobium* were less abundant in the efficient group than in the inefficient group [5]. In the ileum, OTUs assigned to *Bulleidia* and *Saccharopolyspora* were more abundant in the efficient group than in the inefficient group, while one OTU assigned to *Bacillus* was less abundant in the efficient group than in the inefficient group [5]. In the cecum, OTUs assigned to the genera *Dorea*, *Coprococcus*, *Butyrivibrio*, *Lachnospira*, *Sutterella*, and *Anaeroplasma* were more abundant in the efficient group than in the inefficient group, while OTUs assigned to the families *Lachnospiraceae*, *Ruminococcaceae*, and *Erysipelotrichaceae* were more or less abundant in the efficient group than in the inefficient group [5]. In the colon, OTUs assigned to the order *Clostridiales* were more abundant in the efficient group than in the inefficient group, whereas OTUs assigned to the genera *Coprococcus* and *Pirellulaceae* were less abundant in the efficient group than in the inefficient group [5]. Wang et al [48] evaluated the ruminal, duodenal, jejunal, ileal, cecal, colonic, and rectal microbiota and identified microbial differences across the GI tract in crossbred cattle. In this study, *Actinobacteria* and *Patescibacteria* were dominant in the small intestine, while *Ruminococcaceae*, *Rikenellaceae*, and *Bacteroidaceae* were dominant in the large intestine [48]. Although intestinal microbes are less diverse than ruminal microbes, these studies have identified possible intestinal microbiota that can serve as potential biomarkers to represent high-feed efficiency, and their manipulation may be used as strategies to improve feed efficiency in ruminants.

## ASSESSMENT OF FECAL MICROBIOTA

The fecal microbiota in ruminants can affect animal health and food safety. Various factors affect both the fecal microbiota composition and the ruminal microbiota composition; however, the overall fecal microbiota composition differs from the ruminal microbiota composition [49]. Recent studies of the changes in the fecal microbiota due to various factors, such as diet, gender, feed efficiency, and pathogen prevalence, using 16S rRNA gene amplicon sequencing, have improved the understanding in the field of ruminant nutrition.

### Diet and fecal microbiota

Previous studies indicated that fecal microbiota composition differed among cattle fed different levels of dried distillers grains plus solubles (DDGS) [50] and cattle fed different levels of wet distillers grains diets (DG) [51]. Callaway et al [50] indicated that *Acinetobacter* was lower in the 0% DDGS group than in the 25% and 50% DDGS groups in cattle (Table 1). Rice et al [51] showed that *Clostridium*, *Ruminococcus*, *Oscillibacter*, *Hydrogenoanaerobacterium*, *Pseudoflavonifractor*, *Ethanoligenens*, *Selenomonas*, and *Desulfonispora* were more abundant in the 15% DG group than in the 5% DG group, whereas *Parabacteroides* and *Barnesiella* were less abundant in the 15% DG group than in the 5% DG group in cattle (Table 1). Kim et al [52] showed that the fecal microbiota composition of 426 beef cattle was affected by feeding different levels of concentrates. In this study, *Oscillibacter*, *Roseburia*, *Faecalibacterium*, *Coprococcus*, *Blautia*, *Lactobacillus*, *Subdoligranulum*, *Anaerovibrio*, *Prevotella*, and *Bacteroides* were more abundant in the concentrate-based diet group than in the forage-based diet group, whereas *Sporacetigenium*, *Anaerovorax*, *Propionibacterium*, and *Akkermansia* were more abundant in the forage-based diet group than in the concentrate-based diet group (Table 1). Our recent study also indicated that diet greatly affected the fecal microbiota in Hanwoo cattle [53]. In this study, *Romboutsia*, *Paeniclostridium*, and *Turicibacter* were differentially more abundant in Hanwoo cattle fed the late fattening total mixed ration (TMR) diet, while *Akkermansia*, *Bacteroides*, and *Monoglobus* were differentially more abundant in the Hanwoo cattle fed TMR plus oat hay. Diet is a major factor affecting the fecal microbiota composition, and an appropriate diet is necessary to maintain gut health in ruminants.

### Gender and fecal microbiota

To date, little study has been conducted to compare the fecal microbiota between different genders of ruminants. Our recent study compared the fecal microbiota composition between Hanwoo steers and heifers fed the same diet at the same farm [53]. In this study, *Marvinbryantia*, *Coprococcus*, *Alistipes*, and *Ruminococcus* were differentially abundant between Hanwoo steers and heifers under the same dietary condition (Table 1) [53]. The results showed that gender influenced the fecal microbiota composition of Hanwoo cattle. Different sex hormones could lead to microbial differences between genders because bile acid profiles affecting gut microbiota can be shifted by sex hormones [27]. Consideration of the diet×gender interaction may be useful for maintaining gut health in ruminants.

### Feed efficiency and fecal microbiota

Similar to findings for the rumen, there is a possible link between feed efficiency and the fecal microbiota in cattle. Some studies indicated that the fecal microbiota composition differed between high- and low-feed efficiency cattle [54,55]. Lourenco et al [54] compared fecal microbiota between efficient and inefficient Angus steers during the feedlot-finishing stage. Their results showed that *Ruminococcaceae* and *Clostridiaceae* were decreased in inefficient Angus steers, whereas *Peptostreptococcaceae* and *Turicibacteraceae* were increased in efficient Angus steers during the feedlot-finishing stage (Table 1). Welch et al [55] showed that *Ruminococcaceae*, *Rikenellaceae*, and *Christensenellaceae* were more abundant in efficient Angus steers than inefficient Angus steers (Table 1). Our recent study also noticed differences between high- and low-feed efficiency groups of Hanwoo cattle; specifically, *Paeniclostridium* and *Romboutsia* were less abundant in efficient Hanwoo steers than in inefficient Hanwoo steers [56]. Differentially abundant taxa between the two extreme groups may be used as potential biomarkers of high-feed efficiency in cattle. Manipulation of the fecal microbiota composition may be a strategy to improve feed efficiency in ruminants.

### Pathogens and fecal microbiota

Pathogenic *Escherichia coli* (*E. coli*) O157:H7 is commonly found in cattle feces and can infect humans through contaminated food [57]. Although most cattle shed low numbers of *E. coli* O157:H7 in their feces, some supershedder cattle produce a great number of *E. coli* O157:H7 in their feces. Kim et al [10] indicated that the fecal microbiota composition was different between high and low *E. coli* O157:H7 prevalence and enumeration groups of beef cattle, suggesting that manipulation of the fecal microbiota composition may be a strategy to reduce *E. coli* O157:H7 shedding. The addition of corn wet distillers grains with solubles to the diet of cattle increased *E. coli* O157:H7 in the bovine feces [58], whereas the addition of soybean meal decreased *E. coli* O157:H7 in the bovine feces [59]. These studies indicate that dietary interventions can be used as strategies to reduce *E. coli* O157:H7 prevalence and enumeration in cattle feces.

## CONCLUSION REMARKS

In recent nutritional studies, 16S rRNA gene amplicon analysis has been increasingly used to expand the knowledge of the GI microbiota, particularly the ruminal microbiota, and the influencing factors, such as diet, additives, breed, gender, feed efficiency, methane production, and heat stress, in ruminants. The GI microbiota that are differentially abundant in high-efficiency ruminants may be used as potential biomarkers for improving animal productivity. Some heat-stress-resistant microbes may be beneficial as probiotics in ruminant nutrition to enhance the performance or health of cattle under heat stress [44]. Microbial metabolism can be reconstructed from metagenomic data, and new culture media may be devised to isolate and cultivate novel GI microbes [60]. The use of these novel GI microbes may contribute to enhancing the performance of ruminants. In addition, the GI microbiota in ruminants needs to be considered as a heritable phenotype in future studies [61]. The GI microbiota, particularly the ruminal microbiota, may be incorporated into breeding programs to maximize the number of high-performance ruminants. In order to account for the influence of host species and the geographic region, continuous efforts to assess the GI microbiota in domestic ruminants are needed to improve animal performance or health in future studies.

## Figures and Tables

**Table 1:** Summary of factors affecting gastrointestinal microbiota in ruminants

Factor	Region	Animal	Findings regarding microbial differences	Reference
Diet	Rumen	*In vitro* inoculation of rumen fluid collected from Holstein cows	↑*Bacteroidetes* and ↓*Pyramidobacter* by the addition of different combinations of alkaloids, minerals, vitamins, prebiotics, and tannins	[21]
	Rumen	*In vitro* inoculation of rumen fluid collected from Holstein cows	↑*Methanomassiliicoccus* and ↓*Methanobrevibacter* by addition of grape seed procyanidin	[22]
	Rumen	*In vitro* inoculation of rumen fluid collected from Holstein cows	↑*Prevotella*, *Roseburia* and *Megasphaera* by the inclusion of bakery by-products as a feed ingredient	[23]
	Rumen	Lambs	↑*Prevotellaceae* and *Butyrivibrio* by diet with a low energy level	[24]
	Feces	Beef cattle	↓*Acinetobacter* in the 0% dried distillers grains plus solubles (DDGS) group than in the 25% and 50% DDGS groups	[50]
	Feces	Beef cattle	↑*Clostridium*, *Ruminococcus*, *Oscillibacter*, *Hydrogenoanaerobacterium*, *Pseudoflavonifractor*, *Ethanoligenens*, *Selenomona*s and *Desulfonispora* in the 15% wet distillers grains (DG) group than in the 5% DG group ↓*Parabacteroides* and *Barnesiella* in the 15% DG group than in the 5% DG group	[51]
	Feces	Beef cattle	↑*Oscillibacter*, *Roseburia*, Faecalibacterium, *Coprococcus*, *Blautia*, *Lactobacillus*, *Subdoligranulum*, *Anaerovibrio*, *Prevotella* and *Bacteroides* in the concentrate-based diet group ↑*Sporacetigenium*, *Anaerovorax*, *Propionibacterium* and *Akkermansia* were more abundant in the forage-based diet group	[52]
Breed	Rumen	Brown Hanwoo cattle and Jeju black cattle	↑*Ruminococcus* in brown Hanwoo cattle than in Jeju black cattle	[26]
	Rumen	Angus, Charolais, and Kinsella composite hybrid cattle	↑*Bacteroidetes* and *Synergistetes* in Charolais cattle than in the other breeds ↑*Spirochaetes*, *Fibrobacteres*, *Verrucomicrobia*, *Lentisphaerae*, *Tenericutes* and *Chloroflexi* in Kinsella composite hybrid than in the other breeds	[25]
Gender	Rumen	Angus, Charolais, and Kinsella composite hybrid cattle	↑Archaea and ↓Bacteria in bulls than in steers and heifers ↓Archaea and ↑Bacteria in steers than in bulls and heifers	[27]
	Rumen	Tibetan goats	↑*Fibrobacter*, *Ruminococcus*_1 and *Pyramidobacter* in female Tibetan goats than in male Tibetan goats	[28]
	Feces	Hanwoo cattle	↑*Marvinbryantia* and *Coprococcus* in heifers than in steers ↑*Alistipes* and *Ruminococcus* in steers than in heifers	[53]
Marbling	Rumen	Hanwoo cattle	↑*Oscillospira* and *Paludibacter* in the high-marbling score group ↑*Olsenella* in the low-marbling score group	[39]
Heat stress	Rumen	Hanwoo cattle	↑*Prevotellaceae*, *Lactobacillaceae* and *Succinivibrionaceae* in response to short-term heat stress ↓*Ruminococcaceae*, *Desulfovibrionaceae*, *Anaerolineaceae*, and *Pirellulaceae* in response to short-term heat stress	[42]
Feed efficiency	Rumen	Steers	↑*Firmicutes*, *Lachnospiraceae*, *Veillonellaceae* and *Acidaminococcus* in the high-efficient group ↑*Anaerovibrio* in the low efficient group ↑Operational taxonomic units (OTUs) assigned to *Prevotella* in the high efficient group	[34]
	Rumen	Angus, Charolais, and Kinsella composite hybrid cattle	↑*Firmicutes*, *Succiniclasticum*, *Moryella* and *Blautia* in the high efficient group in Charolais cattle ↑*Butyrivibrio* and *Desulfovibrio* in the high efficient group in Kinsella composite hybrid cattle ↑*Shuttleworthia*, *Desulfobulbus* and *Mitsuokella* in the low efficient group in Kinsella composite hybrid cattle	[25]
	Rumen	Angus, Charolais, and Kinsella composite hybrid cattle	↑Ratio of *Firmicutes* to *Bacteroidetes* in the high efficient group	[27]
	Rumen	Beef cattle	↑OTUs assigned to Pr*evotellaceae*, *Spirochaetaceae*, *Paraprevotellaceae*, *Veillonellaceae* and *Lachnospiraceae* in the high efficient group in steers ↑One OTU assigned to *Victivallaceae* in the high efficient group in heifers ↓*Prevotellaceae* and *Fibrobacteraceae* in the high efficient group in heifers	[35]
	Small intestine	Steers	↑OTUs assigned to *Butyrivibrio* in the high efficient group in the jejunum	[45]
	Small intestine	Angus heifers	↑*Lachnospiraceae*, *Ruminococcaceae* and *Christensenellaceae* in the high efficient group in the duodenum ↑*Lachnospiraceae* in the high efficient group in the jejunum ↑*Ruminococcaceae* and *Christensenellaceae* in the high efficient group in the ileum	[4]
	Small intestine	Angus and crossbred steers	↑OTUs assigned to *Lachnospiraceae*, *Ureibacillus*, *Bacillus* and *Prevotella* in the low efficient group in the duodenum ↑One OTU assigned to *Lachnospiraceae* in the high efficient group in the duodenum ↑OTUs assigned to *Butyrivibrio*, *Dialister*, *Desulfovibrio*, *Agrobacterium* and *Ochrobactrum* in the high efficient group in the jejunum ↓OTUs assigned to *Mogibacterium*, *Shuttleworthia*, *Lactobacillus*, *Corynebacterium* and *Atopobium* in the high efficient group in the jejunum ↑OTUs assigned to *Bulleidia* and *Saccharopolyspora* in the high efficient group than in the inefficient group in the ileum ↓One OTU assigned to *Bacillus* in the high efficient group in the ileum	[5]
	Large intestine	Angus and crossbred steers	↑OTUs assigned to *Dorea*, *Coprococcus*, *Butyrivibrio*, *Lachnospira*, *Sutterella* and *Anaeroplasma* in the high efficient group in the cecum ↑OTUs assigned to *Clostridiales* in the high-efficient group in the colon ↓OTUs assigned to *Coprococcus* and *Pirellulaceae* in the high efficient group in the colon	[5]
	Feces	Angus steers	↓*Ruminococcaceae* and *Clostridiaceae* in inefficient steers ↑*Peptostreptococcaceae* and *Turicibacteraceae* in efficient steers	[54]
	Feces	Angus steers	↑*Ruminococcaceae*, *Rikenellaceae* and *Christensenellaceae* in efficient steers	[55]
